# Role of a Home-visit Nursing Agency in Supporting Patients with Heart Failure on Continuous Catecholamine Infusion: A Case Series Study

**DOI:** 10.24546/0100491788

**Published:** 2024-10-16

**Authors:** TAKEMASA ISHIKAWA, RYOKO SEKIGUCHI, TOMOKO SHIMIZU, YUKA FUKATA, REIKO KANAYA, KUMIKO KATSUMA

**Affiliations:** 1 Nana-r Home-visit Nursing Development Center, Osaka, Japan; 2 Nana-r Home-visit Nursing Station, Osaka, Japan

**Keywords:** Cardiomyopathy, Catecholamines, Heart Failure, Home-visit nursing

## Abstract

**AIM:**

This study aimed to discuss the role of home-visit nurses in managing continuous catecholamine infusion in patients with heart failure by investigating the outcome of patients and the home-visit nursing intervention.

**METHODS:**

We conducted a retrospective, case series study of eight patients with heart failure who underwent home-based continuous catecholamine infusion between April 2016 and March 2024. Data including the patients’ demographics, the duration of continuous catecholamine infusion, the frequency of nursing and emergency nursing visits, and patients’ endpoints were collected.

**RESULTS:**

The median age of the patients was 68.5 (interquartile range: 51.8–80.0) years and 75% were men. The most common diagnosis requiring home-based catecholamine infusion was dilated cardiomyopathy. The median duration of continuous catecholamine infusion in the patients was 58.0 days. The median frequency of nursing visits was 8.4 times each week. Forty-five emergency nursing visits occurred, and the most common reason for these visits was managing infusion device malfunctions. Among the patients, six died at home, one was hospitalized owing to fatal arrhythmia, and one withdrew from continuous catecholamine infusion.

**CONCLUSION:**

This study shows the complexities of providing home-based care for patients with heart failure requiring continuous catecholamine infusion. Most patients with heart failure were able to spend the rest of their lives at home, despite the challenges of managing such a treatment outside the hospital. Our findings indicate the need for early intervention, multidisciplinary collaboration, and the development of home care protocols to optimize treatment efficacy and the quality of life of these patients.

## INTRODUCTION

Aging is a factor that contributes to heart failure (HF) (1). In Japan, Shimokawa et al. speculated that there would be a continued increase in the prevalence of HF in Japan, reflecting the effect of an aging population (2). HF is difficult to cure. Blumer et al. reported that 53.3% of patients with a history of hospitalization for HF were readmitted within 1 year, and among them, 15.5% died within 180 days (3). Among those living with HF, withdrawing continuous catecholamine infusion is difficult in some patients. Even in patients with HF who cannot stop continuous catecholamine infusion, some individuals successfully live at home (4). The implementation of continuous catecholamine infusion at home in patients with severe HF is superior regarding healthcare costs compared with in-hospital treatment (5). However, continuous catecholamine infusion at home is not feasible for all patients.

In managing HF at home, the involvement of multidisciplinary teams is crucial (6). While multidisciplinary teams provide comprehensive care, home-visit nurses have a particularly important role within these teams. Taniguchi et al. highlighted the critical role of home-visit nurses in facilitating the continuation of home care for patients with chronic HF and emphasized their importance in monitoring symptoms and managing disease post-discharge (7). The Guidelines by the Japanese Heart Failure Society and Japanese Association for Home Care Medicine specify the nursing care required for administering continuous catecholamine infusion at home (8). However, the extent and frequency of these interventions in actual practice remain unclear. This lack of clarity may lead home-visit nursing agencies to hesitate in accepting patients, fearing insufficient preparation.

This study aimed to discuss the role of home-visit nurses in managing continuous catecholamine infusion in patients with HF by investigating the outcome of these patients and the intervention of home-visit nurses.

## MATERIALS AND METHODS

### Study design

This investigation was conducted as a retrospective case series study using home-visit nursing records.

### Participants and setting

The study included patients who received continuous catecholamine infusion for HF at home and were under the care of the Nana-r home-visit nursing station between April 2016 and March 2024. These participants were identified through electronic nursing records, ensuring a comprehensive overview of the patient population under consideration.

### Data collection

Data were systematically extracted from electronic nursing records, and we specifically focused on nursing documentation. The collected data included patients’ demographics (age, sex, and diagnosis), Ejection fraction, daily life independence level, the independence degree of daily living for the demented elderly, certification of needed long-term care, presence of cohabiting family members, utilization of care services, the duration of continuous catecholamine infusion, the frequency of nursing visits per week and emergency nursing visits per week, including their timing and reasons, and patient endpoints (death, hospitalization, and cessation of catecholamine infusion).

### Data analysis

The collected data were summarized in a table to show the characteristics of the study population, the nature of nursing care provided, and the outcomes observed. Additionally, a survival curve was constructed based on the outcomes occurring within one year following the initiation of home-visit nursing.

### Ethical considerations

The study was approved by the Ethics Review Committee of Japanese Association for Home Care Medicine (number 2024-01). An opt-out consent process was implemented to respect patients’ autonomy and privacy. Information regarding the study was made available on the institution’s website, allowing individuals to decline participation. Verbal explanations were provided for patients who could be directly contacted, followed by obtaining their consent to participate in the study.

## RESULTS

### Demographics of patients with home-based catecholamine infusion

**Figure 1:**
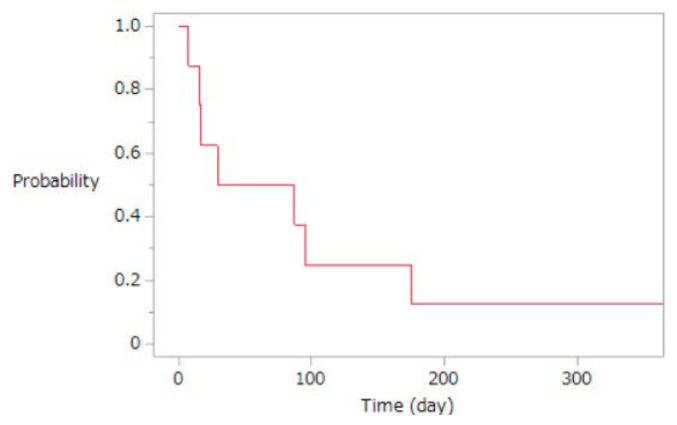
One-year survival curve with ongoing home-visit nursing for patients with heart failure receiving continuous catecholamine infusion

We identified eight patients who underwent home-based continuous catecholamine infusion during the study period (Table I). The primary diagnoses necessitating home-based catecholamine infusion were dilated cardiomyopathy excluding ischemic cardiomyopathy. The median duration of continuous catecholamine infusion in the patients was 58.0 (IQR: 15.5–155.0) days. Figure 1 shows a survival curve for the first year following home-visit nursing initiation. Nursing visits occurred at a median frequency of 8.4 times (IQR: 3.7–15.8) per week, and emergency nursing visits had a median frequency of 0.7 times (IQR: 0.1–1.4) per week. Case 5 started vericiguat 15 months after the initiation of home visits, along with home rehabilitation. The patient expressed a desire to discontinue catecholamine infusion, which allowed for the gradual reduction and eventual cessation of catecholamine infusion.

### Overview of emergency nursing visits

**Figure 2:**
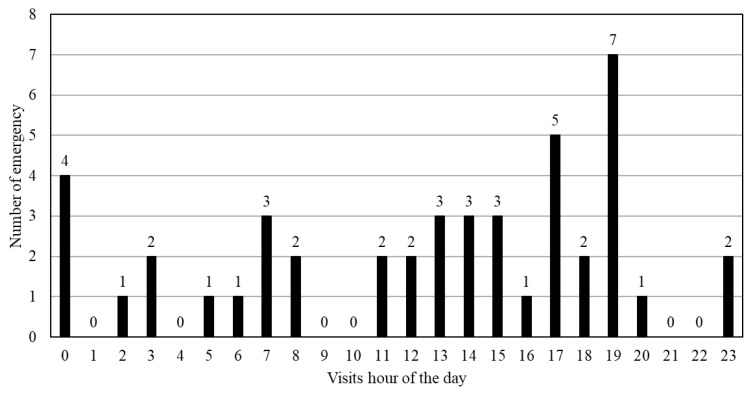
Hourly distribution of emergency nursing visits in patients with heart failure receiving continuous catecholamine infusion

During the study period, 45 emergency nursing visits occurred in patients receiving home-based continuous catecholamine infusion. The distribution of these visits according to the reason is shown in Table II. The most common reason for emergency visits was managing infusion device malfunctions, which accounted for approximately half of all visits, including responding to infusion pump alarms and addressing equipment failure. Our agency does not have clinical engineers. We manage infusion pump issues with pharmacists and medical device companies. Patients and families are instructed on handling alarms and provided with emergency contact information. Home-visit nurses always provide initial instruction and verification, while pharmacists, device suppliers, and hospital nurses also participate in the education process. The next most common reason was the management of distress symptoms (e.g., dyspnea and fatigue). Additionally, the timing of emergency visits was analyzed to understand when patients most frequently required urgent care (Figure 2).

This survival curve represents the outcomes over the one year following the initiation of home-visit nursing for patients with heart failure receiving continuous catecholamine infusion (n = 8). The curve is limited to one year because only one patient survived beyond 175 days, up to 658 days, and thus extending the curve beyond one year would not provide additional meaningful insights.

This figure illustrates the hourly distribution of emergency nursing visits for eight patients with heart failure on continuous catecholamine infusion. The data encompass a total of 44 emergency visits recorded.

## DISCUSSION

### Demographics of patients with home-based catecholamine infusion

This study detailed the demographics and clinical management of patients undergoing home-based continuous catecholamine infusion for HF. Notably, dilated cardiomyopathy was diagnosed in half of the cohort, which suggested its prevalence as a major condition requiring continuous catecholamine infusion. This finding is supported by a study by Heilbrunn et al. (9). Our study’s predominance of male patients is in line with existing epidemiological data on HF and is further corroborated by a study performed by Grupper et al. (10). A finding in our study is that 75% of the patients died at home. This finding suggested that managing patients with continuous catecholamine infusion at home is feasible with robust home-visit nursing support. This region has the highest availability of home-visit nursing agencies in the country (11), providing a supportive environment for such care. Despite the complexities of their treatment, patients with severe HF can stay at home. This aligns with global practices where home-based management is common, confirming that appropriate support structures make home care feasible for such patients (4, 5). This finding is consistent with a study by Martens et al. and contributes to the evidence base that supports the feasibility of home care for patients in the terminal stages of HF (5). However, notably, the median duration of catecholamine infusion in our study was shorter than that reported in previous studies (4, 5). This shorter duration may be influenced by various factors, including delayed discharge, the progression of the patient’s disease, treatment protocols, and patient compliance. Further research is needed to explore these factors in detail to better understand their impact and optimize treatment strategies.

The median frequency of nursing visits, which was 8.4 per week, indicates the demanding nature of the nursing care required for these patients. This level of nursing support, which is higher than that in patients with HF in the general population in Japan, is essential for addressing the multifaceted requirements of patients on continuous catecholamine infusion, including routine monitoring and the management of emergent complications. Only about 2.5% of HF patients in Japan receive home-visit nursing services more than once per day (12). Our data suggest that the higher frequency of home-visit nursing may be, in part, a result of the limitations imposed by Japan’s healthcare and long-term care insurance systems for end-stage heart failure, as outlined in the JCS/JHFS 2021 Statement on Palliative Care in Cardiovascular Diseases (13). These restrictions, which limit the simultaneous use of home-visit nursing and care services like home-visit care, may force home-visit nursing to take on additional responsibilities. If these insurance regulations were adjusted, there could be greater potential for task-sharing between home-visit nursing and care services, allowing for a more balanced distribution of roles and possibly reducing the frequency of nursing visits.

The occurrence of an emergency hospitalization due to lethal arrhythmia in one patient in our study emphasizes the necessity of vigilant monitoring and emergency response preparedness in the management of catecholamine therapy because arrhythmia is a side effect of catecholamine infusion (14). This incident accentuates the vital role of home-visit nursing in delivering comprehensive care and managing acute complications. However, the successful withdrawal of catecholamine infusion in one patient in our study indicates that, despite the general association of home-based catecholamine therapy with end-stage HF (4), tapering or cessation of the therapy may be feasible in some patients, and is contingent upon their clinical status.

Our findings indicate the importance of careful monitoring and frequent home visits for patients with HF receiving continuous catecholamine infusion. Managing severe HF at home depends on regular and intensive nursing care, as seen in the successful management of patients in this study. The need for close monitoring to address potential complications, such as arrhythmias, underscores the role of consistent home nursing care in maintaining patient safety.

### Overview of emergency nursing visits

The results suggest the necessity for continuous, 24-hour management of devices and alleviation of symptoms in end-stage HF.

This study showed that a considerable proportion of emergency nursing visits was for issues related to infusion device malfunctions. This finding suggests that extensive education on the operation and troubleshooting of these devices is required for patients and their families. Managing severe HF patients who require continuous inotropic infusion at home necessitates a well-coordinated multidisciplinary healthcare team. Physicians oversee HF management and palliative care, clinical engineers ensure the proper functioning of infusion devices, and pharmacists manage the safe use and supply of critical medications. This collaboration is essential to provide safe and effective care in the home setting (15). This collaborative model facilitates a comprehensive care strategy that addresses the technical facets of infusion therapy along with the patient’s clinical needs. Additionally, the requirement for interventions to address distress symptoms indicates the need to integrate palliative care principles into the therapeutic plan. Patients with HF frequently encounter distressing symptoms, such as nocturnal awakening due to dyspnea and sleep disturbances, which greatly impair their quality of life (16). To effectively manage these symptoms, a home-visit nursing service capable of providing 24-hour support is essential. Moreover, a multidisciplinary approach is advocated as an effective method to alleviate the discomfort associated with HF (17). This integrated care model requires close collaboration among home-visit nurses, physicians, and palliative care specialists to offer symptom relief and enhance the quality of life in patients in the advanced stages of HF.

### Limitations

While this study provided insights into the management and outcomes of continuous catecholamine infusion in patients with HF at home, several limitations should be acknowledged. First, our findings were based on observational data from a small cohort of eight patients, which may not be representative of the broader population of patients with HF undergoing home-based catecholamine infusion. This small sample size also restricted the statistical analysis of the study, which made generalizing the results to all patients receiving such treatment challenging. Additionally, using only electronic nursing records for data collection likely resulted in the exclusion of critical clinical details. The study was limited by the inability to adequately obtain information on the severity of HF. The descriptive nature of the data analysis provided a summary of the patient’s characteristics and care. However, this approach did not allow for in-depth statistical comparisons or examination of specific causal relationships between variables, such as the direct impact of home-visit nursing on the ability of patients with continuous catecholamine infusion to remain at home and the extent to which home-visit nursing contributed to the success of their home-based care. This study’s findings on the reasons for emergency nursing visits and the timing of these visits offer important preliminary insights. However, the specific reasons for emergency visits (e.g., managing infusion device malfunctions and addressing distress symptoms) highlight the requirement for further research into the effectiveness of current management strategies and the development of new approaches to minimize such emergencies.

While this study contributes to the understanding of home-based care for patients with HF requiring continuous catecholamine infusion, the limitations described above suggest caution in interpreting our findings. Future research with larger, more diverse patient populations and a prospective study design is required to validate and expand upon these observations. Additionally, we could not gather sufficient information on the involvement and support provided by hospitals and other healthcare professionals. Future studies should include detailed information on these aspects to fully understand and improve multidisciplinary collaboration in the care of these patients.

## CONCLUSION

This study of eight patients shows a critical requirement for 24-hour nursing support and emphasizes the complexities and potential emergencies associated with catecholamine infusion and HF symptoms. Notably, the majority of patients with HF were able to spend their final moments at home, despite the challenges of managing such a treatment outside the hospital setting. This outcome suggests the importance of round-the-clock nursing availability in not only ensuring patient safety but also in facilitating home-based end-of-life care. Our findings highlight the necessity for a comprehensive, responsive, home nursing care system that supports patients throughout their treatment and into their final stages of life.

## Figures and Tables

**Table I:** Patient characteristics for heart failure patients on continuous catecholamine infusion

		Case 1	Case 2	Case 3	Case 4	Case 5	Case 6	Case 7	Case 8
Demographics	Age	50s	70s	50s	40s	90s	70s	60s	80s
	Sex	Male	Male	Female	Male	Female	Male	Male	Male

Medical information	Diagnosis	Marfan syndrome (post valve-sparing aortic root replacement)	Dilated cardiomyopathy, Severe mitral regurgitation	Marfan syndrome (post Bentall operation)	Tetralogy of Fallot, Patent Foramen Ovale	Severe tricuspid regurgitation	Dilated cardiomyopathy	Dilated cardiomyopathy	Dilated cardiomyopathy
	Ejection fraction (%)	31	20	N/A	41	N/A	20	19	N/A
	Duration of continuous infusion (days)	87	95	17	175	658	15	7	29
	Catecholamine γ dosage	3	6	1	3	3	5	7	3
	Home oxygen therapy (mL/min)	3.0	None	None	2.0	None	3.0	3.0	0.5

Daily life independence	Daily life independence level*	B1	B2	A2	B1	A2	B2	C2	C2
	Independence degree of daily living for the demented elderly**	No dementia symptoms	No dementia symptoms	No dementia symptoms	No dementia symptoms	II	I	No dementia symptoms	No dementia symptoms
	Certification of needed long-term care	None	Care level 5	None	None	Care level 2	None	Care level 4	Care level 1

Living situation and services	Co-resident	Spouse, Child	Live alone	Spouse	Spouse	Child	Spouse	Spouse, Child	Spouse
	Use of care services	None	Home-visit care (once daily)	None	None	Home-visit rehab (once a week)	None	Welfare equipment	Welfare equipment Home-visit care (twice daily)
	Nursing visit (per week)	8.4	8.3	5.4	2.8	3.1	15.9	17.0	15.7
	Emergency nursing visit (per week)	0.5	1.0	0.0	0.2	0.1	1.4	4.0	1.2

Endpoint		Death at home	Death at home	Emergency hospitalization	Death at home	Cessation of infusion	Death at home	Death at home	Death at home

* A measure of daily life independence, with each rank further divided into two levels. A: Independent indoors, needs help to go out. B: Mostly in bed, needs help indoors. C: Completely bedridden.

** A measure of daily life independence for elderly with dementia. Each level is further divided into two sub-levels. I: No assistance required for daily living. II: Some to significant assistance required. III: Needs substantial to continuous assistance. IV: Fully dependent on assistance. M: Requires medical care and full assistance.

N/A: Not available. Demographic, medical information, and daily life independence data were collected at the initiation of home-visit nursing.

**Table II:** Frequency and reasons for emergency nursing visits

Reason of emergency nursing visits	n (%)
Managing infusion device malfunctions	23 (51.1%)
Management of distress symptoms	8 (17.8%)
Falls	4 (8.9%)
Respiratory arrest	4 (8.9%)
Assistance with dressing	2 (4.4%)
Vomiting	1 (2.2%)
Unknown	3 (6.7%)

Total	45 (100%)

The frequency and reasons for emergency nursing visits among patients with heart failure on continuous catecholamine infusion.
